# The impact of civil conflict on infant and child malnutrition, Nigeria, 2013

**DOI:** 10.1111/mcn.12968

**Published:** 2020-02-11

**Authors:** Embry Howell, Timothy Waidmann, Nancy Birdsall, Nikhil Holla, Kevin Jiang

**Affiliations:** ^1^ Urban Institute Health Policy Center USA; ^2^ Center for Global Development NW Washington, D.C. USA; ^3^ Booz Allen Hamilton McLean, VA USA; ^4^ Baylor College of Medicine Texas Medical Center Houston Texas USA

**Keywords:** child health, conflict, malnutrition, Nigeria, war, wasting

## Abstract

The new millennium brought renewed attention to improving the health of women and children. In this same period, direct deaths from conflicts have declined worldwide, but civilian deaths associated with conflicts have increased. Nigeria is among the most conflict‐prone countries in Sub‐Saharan Africa, especially recently with the Boko Haram insurgency in the north. This paper uses two data sources, the 2013 Demographic and Health Survey for Nigeria and the Social Conflict Analysis Database, linked by geocode, to study the effect of these conflicts on infant and young child acute malnutrition (or wasting). We show a strong association in 2013 between living close to a conflict zone and acute malnutrition in Nigerian children, with larger effects for rural children than urban children. This is related to the severity of the conflict, measured both in terms of the number of conflict deaths and the length of time the child was exposed to conflict. Undoubtedly, civil conflict is limiting the future prospects of Nigerian children and the country's economic growth. In Nigeria, conflicts in the north are expected to continue with sporadic attacks and continued damaged infrastructure. Thus, Nigerian children, innocent victims of the conflict, will continue to suffer the consequences documented in this study.

**Key messages**

- The suffering of small children is often an unrecognized outgrowth of civil conflict.
- The effect of conflict on child malnutrition in Nigeria has not been studied, even though the country is one of the most conflict‐prone in the world.
- We find that infant and young children exposed to conflict in Nigeria are much more likely to suffer from acute malnutrition.
- The effects are more extreme when they live close to the conflict or if the conflict persists over time.
- More should be done to address the nutritional needs of young children in conflict zones.

## 1 INTRODUCTION

The new millennium brought renewed attention to improving the health of women and children, resulting in the adoption of the Millennium Development Goals for 2015 and, more recently, the Sustainable Development Goals for 2030 (World Health Organization, 2015). In establishing these goals, the nations of the world came together to attempt to reduce hunger, infant mortality, and child mortality, with specific targets worldwide. Developing countries, especially Sub‐Saharan African countries, face particular challenges meeting these ambitious goals.

In this same period, direct deaths from conflicts have declined worldwide, but civilian deaths associated with conflicts have increased (Dupuy & Rustad, 2018; Kruk, Freedman, Anglin, & Waldman, 2010; World Health Organization, 2015). One study estimates that in 2016, 59% of the world's children lived in conflict‐affected countries (Bahgat, Dupuy, Ostby, Rustad, Strand, & Wig, 2018). Armed conflicts are more likely to occur in poor countries with weak states, particularly in sparsely populated rural areas and in places where there is competition for land and water (Blattman & Miguel, 2010).

Numerous studies have shown the negative health impact of such conflicts on civilians, particularly children. Table 1 summarizes findings from 17 empirical studies, including one pan‐African study of 35 countries, 11 African country‐specific studies, and five non‐African country‐specific studies. All concern the impact of armed conflict on maternal and child health. Although no study is completely free from methodological limitations, the combined results suggest what is intuitively obvious that—across many countries—there is a substantial negative effect on maternal and child health from war and conflict. The most recent study, and most methodologically rigorous, shows a clear relationship between African conflicts and infant mortality in 35 countries over a substantial time period (Wagner, HeftNeal, Bhutta, Black, Burke, & Bendavid, 2018). However, caution is needed because even the most rigorous studies show a correlation that may or may not be causal. For example, one study in Ethiopia concluded that although there was a correlation between conflict and child health, after controlling for drought conflict events did not have a significant effect on child health (Delbiso, Rodriguez‐Llanes, Donneau, Speybroeck, & Guha‐Sapir, 2017). In addition, the size of effects cannot be generalized across the studies because of substantial methodological differences. For example, the child health outcomes in these studies are quite varied, including various measures of poor nutritional status, such as small weight‐for‐age, height‐for‐age, or weight‐for‐height (“wasting” or “acute malnutrition”). Counting the number of conflicts and the characteristics of those conflicts is challenging and different data sources tally such events in different ways, each with potential biases (Johnson, Spagat, Gourley, Onnela, & Reinert, 2008). The most common limitation among the studies cited in Table 1 is that conflict exposure was generally measured by residence in a country, region, province, or district where conflict was underway for a period of time, with the exception being the Wagner et al. study cited previously and the Kinyoki et al. (2017) study. A particular mother's or child's exposure to conflict and the intensity of that person's exposure, as distinct from any other resident of the same geographic area, generally was not measured. Other limitations of one or more studies include use of aggregate data from a variety of surveys, small sample sizes, and a lack of adjustment for maternal, child, or household characteristics.

**Table 1:** Summary of findings from empirical studies of the effect of conflict on mothers and children

Citation	Countries	Dates of study	Key findings	Study limitations
African countries				
Akresh, Lucchetti, and Thirumurthy (2012)	Eritrea and Ethiopia	2002	War‐exposed children had 22% lower height‐for‐age scores than non‐war‐exposed children.	Conflict exposure measured by living in a conflict region.
Avogo and Agadjanian (2010)	Angola	2004	Child mortality was twice as high if the child experienced war‐related forced migration in the previous year.	Sample limited to two urban municipalities.
Coghlan et al. (2006)	DRC	2003–2004	The under 5 mortality rate was twice as high in health zones reporting violence as in health zones not reporting violence.	5 million people could not be included in population sampled due to security issues.
Dabelen and Paul (2014)	Cote d'Ivoire	2002–2008	Households in departments with at least one conflict event were more food insecure. Outcomes were worse for households with women and children.	No measure of intensity of conflict exposure (e.g., duration or deaths).
Delbiso et al. (2017)	Ethiopia	2000–2013	Weight‐for‐height (wasting) was lowest in drought‐prone areas. Conflict events did not have a significant impact on wasting after controlling for drought.	Used aggregate data from a variety of surveys and populations; no measure of conflict intensity.
Guha‐Sapir, van Panhuis, Degomme and Teran (2005)	Angola, DRC, Ethiopia, Sudan	2000–2004	In all countries, the conflict zones experienced higher child mortality and acute malnutrition than nationally.	Used aggregate data from a variety of surveys and populations; not nationally representative.
Kinyoki et al. (2017)	Somalia	2007–2010	Children exposed to recent conflict had a higher risk of wasting (OR = 1.38). Risk of stunting was similar. Children exposed to longer conflicts had higher risk.	No adjustment for intensity of conflict (e.g., number of deaths).
Lindskog (2016)	DRC	2007–2014	Postneonatal mortality was highest where conflict events and deaths were extremely high. Neonatal mortality was not affected by conflict levels.	Conflict levels measured at the province level.
Minoiu and Shemyakin (2014)	Cote d'Ivoire	2002–2008	Children in conflict zones had significantly lower height‐for‐age scores.	Conflict exposure measured by living in a conflict region; no measure of conflict intensity.
Namasivayam, Arcos, Castro and Chi (2017)	Uganda	1988–2011	Women in the conflict zone had lower rates of contraception use and institutional delivery but higher rates of skilled delivery.	Conflict exposure measured by living in conflict zone; no measure of conflict intensity.
Verwimp (2012)	Burundi	1998–2007	Children exposed to civil war in their area of residence had a 10% increase in the probability of dying.	Small sample size (*N* = 283, only 75 exposed to civil war).
Wagner et al. (2018)	35 African countries	1995–2015	The risk of infant death greatly increased when the family lived near an armed conflict. Infant deaths related to conflict were over three times the number of direct deaths from conflicts.	Lack of adjustment for migration.
Other countries				
Ascherio et al. (1992)	Iraq	1991	Age‐adjusted child mortality rates were three times as high after the Gulf War in 1991 than before the war.	No measure of intensity of conflict exposure.
Guerrero‐Serdan (2009)	Iraq	2000–2006	Children born in the highest intensity conflict provinces during the Iraq war of 2003 were .8 cm shorter than children born in low conflict provinces.	Conflict exposure measured by living in a conflict region or district.
Mashal et al. (2008)	Afghanistan	2006	Internal displacement was associated with low weight‐for‐age in children.	Sample restricted to two urban districts; no direct measure of conflict exposure.
Savitz et al. (1993)	Vietnam	1960–1988	Postneonatal mortality increased significantly during the war. Neonatal mortality and childhood mortality did not change significantly during the war. There was no increase in infant or child mortality after the war.	No measure of intensity of conflict exposure.
Skokic, Muratovic and Radoja (2006)	Bosnia and Herzegovina	1988–2003	Perinatal and maternal mortality were higher during the war, mainly due to limited access to health services.	Sample restricted to mothers delivering in one canton. No adjustment for maternal or household characteristics.

Armed conflict is prevalent in Sub‐Saharan Africa. Nigeria is among the most conflict‐prone countries in the region, experiencing the highest number of conflict‐related deaths of all Sub‐Saharan African countries in many of the years since 2000 (Armed Conflict Location & Event Data Project [ACLED], n.d.). Since 2000 when civilian government took hold, Nigeria has experienced conflict in the Niger Delta around control of oil fields and oil wealth; conflicts in many parts of the country, particularly in the “middle belt,” concerning competition for land, often with ethnic and religious affiliation underpinnings; and more recently (since 2011) the Boko Haram insurgency in the north (Project Ploughshares, n.d.). One data source documents 32,942 deaths from social violence in Nigeria during the period 1998 to 2014. Of these, 42% were due to the Boko Haram insurgency (all since 2009). Another 45% were communal/ethnic/religious or herder/farmer conflicts. The remainder were due to state or political violence (Nigeria Social Violence Project, n.d.).

According to the Social Conflict Analysis Database (SCAD), the number of conflicts in Nigeria varied from 30 in 2008 to 114 in 2013 (Salehyan et al., 2012). Most (77.7%) conflicts were armed conflicts. Conflict in the period peaked in 2013 due to the Boko Haram insurgency. There are variations in the number of deaths per year, from 844 in 2008 to 2,350 in 2013. In 2013, all but three of the top 20 deadliest conflict events had Boko Haram involvement. As an example of these horrific events, in October, 2013 “Dozens of gunmen on motorbikes and in pick‐up trucks attacked a village in the local government area of Bama. The assailants shot dead 27 people and injured another 12. They also razed some 300 homes, stole motorbikes, cars, livestock and money.” (Jackson, 2013). Much of the violence in 2013 was concentrated in the northeast, near Maiduguri, the capital of the Borno state.

## 2 METHODS

### 2.1 Conceptual framework

**Figure 1:**
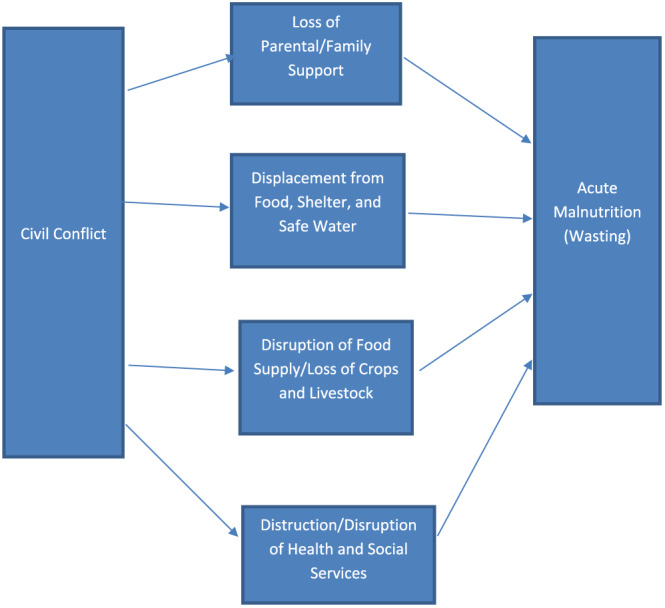
Conceptual framework for impact of conflict on child acute malnutrition [Colour figure can be viewed at wileyonlinelibrary.com]

The purpose of this study is to add to existing literature by documenting the effect of conflict on child malnutrition in Nigeria by improved empirical methods. Figure 1 provides a conceptual framework for how civil conflict may affect child nutrition. Child malnutrition can result from loss of parental or family support, including the loss of farms or herds. Civil conflict can lead to many other factors that affect a child's nutritional status, such as displacement from food, shelter, and safe water (leading to malnutrition, diarrheal disease, and reduced resistance to other diseases) and disruption in the food supply chain and destruction or disruption of health and social services that may prevent access to life‐saving treatments. In addition, the destruction or disruption of health and social services providing life‐saving treatments may occur in the locations where such services are most needed (Pike, Straight, Oesterle, Hilton, & Lanyasunya, 2010; Safeguarding Health in Conflict Coalition, 2017). In particular, aid projects, shown to be effective at reducing infant mortality in Nigeria (Kotsadam, Ostby, Rustad, Tollefsen, & Urdal, 2018), may not be located in the most conflict‐prone areas. Effects may be different for urban and rural children, given differences in the supply of food and health and social services, among other factors.

### 2.2 Data sources

We used two data sources, linked together by geocode. The first is the 2013 Demographic and Health Survey (DHS) for Nigeria, one of hundreds of DHS surveys that have been conducted in 90 developing countries since 1985. The DHS assesses the nutrition, health, and socioeconomic characteristics of a nationally representative sample of women and their children (Demographic and Health Surveys, n.d.). The DHS is implemented by ICF International and is funded by the United States Agency for International Development with contributions from other donors. The DHS data set has been thoroughly edited, cleaned, and documented by ICF. The public use file contains weights that account for the complex multilevel sample design, and these are used in this paper to construct estimates from the sample data.

The second data source is the SCAD (Salehyan et al., 2012), a database compiled from reports of conflicts reported by two wire services, the Associated Press and Agence France Presse. Both the DHS and the SCAD include geocode data in the form of longitude and latitude coordinates; in the case of DHS, these codes identify the location of the interview (the centroid of cluster for the household), and in the case of SCAD, they identify the location of the conflict. The two data sources (SCAD and DHS) were linked using this geocode. Our data set consists of a record for each Nigerian child in the survey and all conflicts that were within a predefined radius around the child's household at the time of the survey. We eliminated conflict events with no deaths.

### 2.3 Outcome and control measures

All data on outcomes derive from the DHS survey conducted in 2013. The DHS is an in‐person interview with mothers. It asks her questions about her health and fertility and that of members of her household. In particular, for this study, she reports the birth dates for each of her children, and children under 5 are measured by the interviewer for height and weight. The survey responses are used to construct the outcome measure (acute malnutrition) for each child under age 5 in the survey, as follows:

The *z*‐score for weight for height is the number of standard deviations above or below the norm weight for height for children, as defined by the World Health Organization, multiplied by 100. Acute malnutrition, also called “wasting,” is defined as weight for height more than 2 standard deviations below the norm. When a height or weight was missing or out of the expected range (as set by the DHS), the *z*‐score was not available and those children were left out of the analysis. Demographic variables, used as control in the regression models, are defined as follows:

- Child characteristics: birth order, year of birth, gender, and number of months since birth of previous sibling (with the overall average used for first order births)
- Maternal characteristics: age at each child's birth, marital status at time of interview, length of education, and ethnicity (tribe)
- Household characteristics: region, wealth index quintile (constructed from numerous DHS indicators such as housing quality), and household size
- Urban/rural residence: According to the Nigeria 2013 DHS, urban residence is defined as living in a large city (over 1 million people), a small city (over 50,000 people), or a town (not defined by population but by the Nigerian government).

### 2.4 Conflict exposure measures

From the SCAD, we developed two indices of child exposure to conflict, with the assumption that a conflict with a higher number of deaths or that lasted longer would have more serious consequences for the child's nutritional status. Thus, conflict exposure is measured by all conflict‐related deaths and by length of exposure during the year preceding the interview. In addition, we assumed that a conflict that was further away would have less consequence for a child's health. The number of conflict deaths is weighted inversely by distance of the conflict to the mother's home at the time of the interview. For example, a death within 1 km of the home has a weight of 1 and a death within 2 km has a weight of.5. Deaths to conflicts outside a 10‐km radius for urban children and a 25‐km radius for rural children are given a weight of zero. A child can be exposed to more than one conflict in a given period, and if so, all deaths are included in the exposure measure. Similarly, a measure of length of exposure in days, also weighted by distance in a similar manner to deaths, is used. In this measure, a child does not necessarily experience the entire duration of the conflict; only those days with one or more conflicts within the child's exposure period are counted.

### 2.5 Data limitations

The DHS has a major limitation for the purposes of this study, in that location data (which are merged with the SCAD) are collected at the time of the survey. However, thousands of people have been displaced from their homes during the Nigerian civil conflicts of the study period (Project Ploughshares, n.d.). Consequently, when a mother is interviewed, she may not reside at or near the place where she gave birth or where a particular child lived during the exposure period. This inserts a substantial measurement bias in the conflict exposure data. It is not clear whether the bias is in a favourable or unfavourable direction. For example, people who are better off (and with better outcomes) may have a better ability to move, but those who are worse off (and with poorer outcomes) may be more motivated to move, especially persecuted ethnic groups. In the first circumstance, the bias would be in the direction of finding an effect of the impact of conflict and vice versa for the second circumstance. This problem with using DHS data for the analysis of conflict effects was noted in the first DHS study of this topic (Savitz, Thang, Swenson, & Stone, 1993) and continues today. A second limitation of these data is that distance to a conflict is measured relative to the geographic centroid of a survey cluster rather than to the precise location of the mother's home (clusters are generally small geographic areas.) This introduces random measurement error in the key independent variable, which tends to bias coefficient estimates towards zero, reducing the likelihood of finding a significant effect.

There are several large databases that tabulate the number and location of conflicts in Africa and other countries, as well as the type and length of the conflict and the number of deaths associated with it. One of the most often used is the ACLED (Raleigh, Linke, Hegre, & Karlsen, 2010). Both the SCAD and the ACLED document conflicts in Nigeria over the study period. However, one major difference between the data sets is the sources for reports of conflicts, locations, and conflict deaths. For the SCAD, these come from only two sources, the Associated Press and Agence France Presse news wires. In contrast, the ACLED uses up to 50 local, regional, national, and continental media sources. Not surprisingly, the ACLED documents about twice as many conflict deaths as the SCAD. We chose the more conservative data source in terms of numbers of conflicts and conflict deaths, which likely underreports small localized conflicts but is likely more consistent over time.

## 3 RESULTS

Table 2 shows the characteristics of the study group, separately for urban and rural children. The study group is characterized by children in large families (average family size of 6.6 in urban areas and 7.4 in rural areas). In addition, rural residents are much poorer than urban residents. Only 3.0% of urban residents are in the poorest quintile compared with 33.5% of rural residents. Rural residents are concentrated in the northern regions.

**Table 2:** Characteristics of mothers and children in study group: Nigeria, 2013

	Urban	Rural
Child characteristics		
Birth order (continuous)	3.6	4.1
Gender (male)	49.5%	49.5%
Preceding birth interval (months)	37.9	36.4
Mother characteristics		
Age at child's birth (years)	28.4	27.2
Education		
No education	21.1%	61.7%
Primary school	21.6%	18.6%
Secondary school	44.4%	17.4%
Higher than secondary school	12.9%	2.2%
Not married	4.3%	3.4%
Household characteristics		
Household size	6.6	7.4
Wealth		
Poorest	3.0%	33.5%
Poorer	6.6%	30.9%
Middle	16.0%	21.0%
Richer	32.3%	10.9%
Richest	42.1%	3.7%
Ethnicity		
Hausa/Fulani	26.9%	50.2%
Igbo/Ig	23.3%	4.8%
Yoruba	25.4%	4.5%
Other	24.3%	40.5%
Region		
North Central	8.9%	18.0%
North East	11.5%	20.2%
North West	23.4%	41.3%
South East	17.5%	4.2%
South South	8.9%	9.5%
South West	29.8%	6.8%
*N*	8,507	15,961

Source: Nigerian Demographic and Health Survey, 2013.

Using the DHS Statcompiler (n.d.), we found that unadjusted rates of wasting in 2013 for urban and rural Nigerian children in the sample were very high (data not shown). The child wasting rate was 19.2%, with 19.0% for urban children and 19.3% for rural children. The mean *z*‐score was −0.6 (*SD*, 1.5).

Table 3 shows the average exposure of children in the sample to conflicts and conflict deaths. The table shows both absolute numbers of conflicts and conflict deaths as well as the number of deaths weighted by distance from conflict. Keeping in mind the potential location bias discussed above, the data suggest that Nigerian children were exposed to many conflicts during the study period, which took place during the Boko Haram insurgency. An urban child was exposed to an average of 4.32 conflicts in the year prior to the survey, whereas a rural child was exposed to an average of 1.04 conflicts. The average annual number of conflict deaths was 8.94 deaths (urban children) and 2.43 deaths (rural children). After weighting for distance, those numbers become 1.83 deaths for urban children and only 0.14 deaths for rural children. The average length of conflicts was 11.85 days (urban) versus 3.08 days (rural) during the year before the survey. After weighting for distance, this conflict measure is 2.78 days (urban) and 0.16 days (rural).

**Table 3:** Average annual exposure to conflicts: Children ages 0–4, Nigeria, 2013

Average annual exposure to conflicts by type (unweighted)	Urban	Rural
Demonstration^a^ or strike^b^	0.62	0.08
Violent riot^c^	0.13	0.04
Internal governmental violence^d^	1.82	0.46
External governmental violence^e^	1.75	0.45
Average annual total conflicts during exposure period (unweighted)	4.32	1.04
Average annual conflict deaths (unweighted)	8.94	2.43
Average annual length of conflict (days, unweighted)	11.85	3.08
Average annual conflict deaths (weighted)	1.83	0.14
Average annual length of conflict (days, weighted)	2.78	0.16

a Demonstration—Peaceful action directed toward members of another group or government authorities.

b Strike—Engaging in abandonment of workplaces and public facilities.

c Violent Riot—violent action directed towards members of another group or government authorities.

d Internal governmental violence—violence waged against government authorities, by government authorities, or within the government itself.

e External governmental violence—violence waged by nonstate groups without government actors or targets.

Table 4 presents results from four logistic regression analyses of the impact of exposure to conflict on child wasting. Separate models are presented for urban and rural children, as well as separate models for the two conflict measures, conflict deaths and conflict duration. All four regressions show consistently that conflict is positively and significantly associated with child wasting (acute malnutrition). For example, the odds of experiencing wasting are 5% higher for every unit increase in the conflict death index (deaths/km, e.g., one death within 1 km and two deaths within 2 km) in urban areas and 17% higher in rural areas (borderline significance). This is a substantial increase in the odds of wasting for the average child, especially for urban children who are exposed to more conflicts.

**Table 4:** Logistic regression predicting risk of child wasting: Ages 0–4, Nigeria, 2013

Control variables	Conflicts exposure: Weighted deaths		Conflict exposure: Weighted days	
	Urban	Rural	Urban	Rural
Conflict deaths weighted by distance (continuous)	1.05**	1.17***	—	—
Conflict days weighted by distance (continuous)	—	—	1.04**	1.13*
Preceding birth interval (continuous months)	0.999	1.001	1.000	1.001
Birth order (continuous)	1.02	0.97	1.02	0.97
Child gender (male)	1.29**	1.07	1.28**	1.07
Mother's age at child's birth (continuous years)	0.99	1.01	0.99	1.01
Mother's highest education level				
No education (reference)	1	1	1	1
Any primary school	0.99	0.80*	0.97	0.81*
Any secondary school	0.88	0.70**	0.84	0.70**
Higher than secondary school	0.62*	0.58***	0.60**	0.58***
Mother not married	0.98	0.72*	0.98	0.72*
Number of household members (continuous)	0.998	0.999	0.996	1.00
Ethnicity				
Hausa/Fulani (reference)	1	1	1	1
Igbo/Ig	1.39	0.61	1.61***	0.61
Yoruba	1.36	0.51*	1.57*	0.51*
Other	1.57*	0.84	1.75**	0.85
Region				
North West (reference)	1	1	1	1
North Central	0.23**	0.65**	0.23**	0.68**
North East	0.30**	0.97	0.40**	0.99
South East	0.24**	1.02	0.23**	1.04
South South	0.19**	0.67**	0.19**	0.66**
South West	0.23**	0.54**	0.21**	0.54**
*N*	8,507	15,961	8,507	15,961

*Note.* Year of child's birth is also included in the regression.

** *p* < .01.;

* *p* < .05.;

*** *p* < .10 (borderline significance).

It is possible that a lack of access to good nutrition affects all children throughout the weight for height spectrum. For example, children who have an excellent weight for height may lose weight, even though they do not become acutely malnourished (wasted). To examine whether the relationship of conflict to child nutritional status is continuous throughout the weight for height spectrum, we also modelled the continuous *z*‐score. There was no clear cut point, suggesting a gradually declining nutritional status below the norm. This regression also showed a strong and significant relationship between both the number of conflict deaths weighted by distance and the duration of the conflict weighted by distance. For example, using the conflict death measure, for urban areas, a conflict death within 1 km led to a decrease of.059 *SD* from the WHO norm of weight for height for the child's age. These regression results can be found in a separate document (Howell, Waidmann, Birdsall, Holla, & Jiang, 2018).

We conducted several sensitivity analyses. First, to understand the sensitivity of the findings to the treatment of distance, we recalculated the conflict metrics using the squared distance rather than the linear distance. Although coefficient values change (as expected given the change in the scale), all significant coefficients shown in the paper remain statistically significant. Similarly, to control for long duration events, we added dummy variables indicating conflicts lasting between 3 and 12 months and conflicts lasting more than 12 months. In both sets of analyses using different distance weighting methodologies, the values of the coefficients on the conflict indicators changed only slightly and remained significant. Other sensitivity analyses are described in the previously cited document (Howell et al., 2018).

## 4 DISCUSSION

This article has shown a strong association between living close to a conflict zone and acute malnutrition (wasting) in Nigerian children in 2013. This is related to the severity of the conflict, as measured both in terms of the number of conflict deaths and the length of time the child was exposed to conflict. For example, urban children have about 5% greater odds of being wasted for each conflict day or death they are exposed to. Findings for rural children are even stronger with odds ratios of 1.13 or 1.17 depending on the conflict measure. The analysis of *z*‐scores shows that not only the most acutely malnourished children are affected by conflict. The average child moves further below the international norm of weight for height with the increase in conflict intensity.

The finding that urban children experienced more conflicts than rural children was somewhat surprising, but is likely explained by two factors. First, the definition used by the DHS of “urban” included towns as well as cities. In addition, a particular circumstance of the Nigerian conflict in 2013 was that the Boko Haram insurgency took place in and around Maiduguri, the capital of Borno state and a city of over a million people.

Our findings are generally consistent with findings from other countries on the negative impact of conflict on child nutrition. However, these results provide an improved methodology for relating the size, duration, and location of a conflict to child malnutrition. Most previous studies have examined conflicts in a particular region of a country but have not distinguished between how close the conflict was to the mother and child's residence and generally have not examined conflict severity. The exception is the previously mentioned Lancet article (Wagner et al., 2018), but that study did not address acute malnutrition. In addition, previous studies have not examined Nigeria specifically, where conflict has become endemic in certain parts of the country.

There is a lack of good evidence on what works to minimize the profound negative effects of conflict on children. De Jong suggests that conflict should be treated as a public health problem, with primary, secondary, and tertiary interventions (De Jong, 2010). Primary prevention would consist of addressing the underlying causes of conflict (e.g., corrupt governments, lack of education and jobs, the flow of small arms into countries, and increasing drought). Secondary prevention consists of numerous targeted interventions, such as food assistance and “therapeutic feeding” (e.g., using enriched foods) for malnourished children (Maternal and Child Undernutrition Study Group, 2008; Young, Borrel, Holland, & Salama, 2004). Tertiary prevention includes peace‐keeping after the conflict to avoid recurrence as well as rehabilitation of education and health facilities. A recent World Bank report suggests several ways that the Bank could improve its assistance to postconflict countries to address children's health, such as institutional support to Ministries of Health and to local governments, more flexible financing, and more explicit efforts to address the health needs of children postconflict (Bustreo, Genovese, Omobono, Axelsson, & Bannon, 2005). International aid and development projects can provide needed assistance but are often not targeted to the most vulnerable populations (Kotsadam et al., 2018).

## 5 CONCLUSION

Undoubtedly, civil conflict is limiting the future prospects of Nigerian children and the country's economic growth. As pointed out in the recent Global Nutrition Report, malnutrition “is a problem of staggering size—large enough to threaten the world's sustainable development ambitions” (International Food Policy Research Institute, 2015).

Conflicts creating humanitarian crises are forecast for 17 countries around the world for 2018. In particular, in Nigeria, conflicts in the north are expected to continue with sporadic attacks and continued damaged infrastructure (Assessment Capacities Project, 2017). Thus, the children in Nigeria will continue to suffer the consequences documented in this study. This makes a response from civil society, donors, and governments all the timelier and more urgent.

## CONFLICTS OF INTEREST

The authors declare that they have no conflicts of interest.

## CONTRIBUTIONS

EH oversaw the design and implementation of the study and drafted the initial manuscript. TW oversaw the statistical design and data analyses and contributed to the manuscript. NB provided a critical review of the study design and the manuscript. NH and KJ analysed the data and contributed to the manuscript. All authors reviewed and approved the manuscript.
